# Organophosphate Flame Retardants in Shanghai Indoor Dust: A 2016 Historical Baseline of Seasonal Variability, Microenvironmental Profiles, and Human Exposure

**DOI:** 10.3390/toxics14090758

**Published:** 2026-08-26

**Authors:** Jiajun Chang, Li Li, Binghong Cheng, Zhiliang Zhu, Qinghui Huang, Yanling Qiu, Åke Bergman

**Affiliations:** 1 Key Laboratory of Yangtze River Water Environment, College of Environmental Science and Engineering, Tongji University, 1239 Siping Road, Shanghai 200092, China; 2210091@tongji.edu.cn (J.C.); lili253159@163.com (L.L.); zzl@tongji.edu.cn (Z.Z.); qhhuang@tongji.edu.cn (Q.H.); 2 Shanghai Institute of Pollution Control and Ecological Security, 1239 Siping Road, Shanghai 200092, China; 3 Hangzhou Ecological Environment Publicity and Education Information Center, Hangzhou 310020, China; chengbh1987@126.com; 4 Department of Environmental Science and Analytical Chemistry, Stockholm University, 10691 Stockholm, Sweden; ake.bergman@aces.su.se

**Keywords:** organophosphate esters, indoor dust, historical baseline, repeated sampling, seasonal variability, exposure assessment, Shanghai

## Abstract

Organophosphate flame retardants (OPFRs) are additive chemicals that migrate from indoor materials and accumulate in settled dust. This study retrospectively characterized eight OPFRs in floor dust collected in Shanghai during 2016 to establish a seasonally resolved historical reference. A total of 136 residential dust samples were obtained from a convenience panel of 26 homes sampled at approximately two-month intervals; five office and four dormitory composite samples were included only for exploratory microenvironmental comparisons. Sampling, extraction, and LC-MS/MS determination were all completed in 2016. OPFRs were detected in all samples. Median (mean) Σ_8_OPFR concentrations were 1.51 × 10^3^ (4.35 × 10^3^), 2.48 × 10^3^ (3.36 × 10^3^), and 673 (963) ng·g^−1^ in residential, office, and dormitory dust, respectively, and chlorinated OPFRs—particularly tris(2-chloroisopropyl) phosphate and tris(2-chloroethyl) phosphate—dominated. Mixed-effects models accounting for repeated sampling confirmed significant season effects for Σ_8_OPFRs and all frequently detected congeners, with autumn generally lower than spring or winter. Under the mean 2016 concentration scenario, uptake-adjusted total estimated daily intakes were 3.3 ng·kg^−1^·day^−1^ for infants and 0.65 ng·kg^−1^·day^−1^ for adults. Oral administered-dose screening values did not exceed the selected non-cancer or cancer benchmarks. These measurements do not represent current exposure but provide a historical benchmark for method-matched follow-up monitoring.

## 1. Introduction

Organophosphate flame retardants (OPFRs), also termed organophosphate esters, are widely used as additive flame retardants and plasticizers in polymers, furniture, electronic equipment, building materials, and surface treatments. Their use expanded as legacy brominated flame retardants were restricted or phased out [1,2]. Global and Chinese emission inventories indicate continued large-scale production and use, with substantial changes in product applications and regional emissions during the past decade [3,4]. Because OPFRs are generally not covalently bound to product matrices, they can be released through volatilization, abrasion, and material wear and subsequently partition to airborne particles and settled dust [1,5].

Indoor dust is an important integrative reservoir and exposure medium for OPFRs. Multi-country surveys and reviews have documented widespread detection, frequent predominance of chlorinated congeners, and marked variation among buildings and microenvironments [6,7,8,9]. This issue is particularly relevant because people spend most of their time indoors [10], while hand-to-mouth behavior and higher dust intake per unit body weight can increase exposure in young children [6,11]. Toxicological evidence for chlorinated OPFRs varies by congener and endpoint, but experimental and regulatory assessments identify neurotoxicity, reproductive and developmental toxicity, endocrine disruption, kidney and other organ toxicity, and carcinogenic potential as concerns [1,6,12,13]. In 2024, the U.S. EPA concluded that TCEP presents unreasonable human-health risk under specific conditions of use, including neurological, reproductive, developmental, kidney, and cancer effects [12]. These concerns support the need to identify priority compounds and characterize exposure pathways.

Interpretation of temporal change requires historical datasets with well-described sampling designs. Many indoor-dust studies are cross-sectional and therefore cannot distinguish seasonal variability from differences among buildings. Repeated sampling of the same residential panel can characterize intra-annual variation and provide a reference for later method-matched surveys. Reported seasonal patterns are not uniform: some studies have observed higher dust concentrations in colder periods, whereas air-phase concentrations may rise during warmer periods because temperature, ventilation, source use, particle deposition, occupant behavior, and cleaning practices influence indoor compartments differently [14,15,16,17,18,19]. Although the samples in the present study were collected and analyzed in 2016, the complete year-round dataset was not previously published. It is reported now because well-documented pre-2020 measurements are increasingly valuable for evaluating long-term changes in OPFR use and exposure, provided they are treated as a dated scenario rather than a proxy for current conditions.

Seasonally repeated measurements within the same residences remain scarce for OPFRs, as most indoor-dust studies rely on cross-sectional sampling and therefore cannot readily distinguish temporal variability from differences among buildings. To address this gap, the broader purpose of this study was to convert an underused year-round 2016 dataset into a transparent historical benchmark that can support future method-matched monitoring. The integrated objectives were to characterize dated occurrence and congener profiles, quantify within-home seasonal variability, explore source-related and microenvironmental indicators, and reconstruct screening-level dust exposure under the 2016 scenario. We hypothesized that (i) frequently detected OPFRs would vary seasonally within residences and (ii) dust-related exposure would be higher for infants than for adults. Office and dormitory measurements were treated as exploratory profile comparisons rather than a formal microenvironmental hypothesis test.

## 2. Materials and Methods

### 2.1. Chemicals and Reagents

Analytical standards of tris(2-chloroethyl) phosphate (TCEP), tripropyl phosphate (TPrP), tris(2-chloroisopropyl) phosphate (TCIPP), tris(1,3-dichloro-2-propyl) phosphate (TDCIPP), triphenyl phosphate (TPhP), tri-n-butyl phosphate (TBP), tris(2-butoxyethyl) phosphate (TBOEP), and tris(methylphenyl) phosphate (TMPP; commonly termed tricresyl phosphate; CAS 1330-78-5; isomer mixture) were purchased from AccuStandard Inc. (New Haven, CT, USA). Isotope-labelled surrogate and internal standards were obtained from Cambridge Isotope Laboratories (Tewksbury, MA, USA). n-Hexane, acetone, ethyl acetate, and HPLC-grade methanol were purchased from Sigma-Aldrich (Darmstadt, Germany), and Florisil solid-phase extraction (SPE) cartridges (500 mg, 3 mL) were obtained from CNW Technologies GmbH (Düsseldorf, Germany). Chemical properties are summarized in Table S1.

### 2.2. Sampling Design and Dust Collection

Floor dust was collected in Shanghai, China, during 2016. Residences were recruited as a convenience panel through volunteer contacts and were not selected using probability sampling; the panel should therefore not be interpreted as statistically representative of all Shanghai homes. The same 26 homes were scheduled for sampling in January, March, May, July, September, and November. These months were assigned as winter (January), spring (March and May), summer (July), and autumn (September and November). The nominal interval was approximately two months, although exact intervals varied slightly with household availability. The campaign yielded 136 raw residential dust samples. Because not every scheduled sample was available from every home and one home contributed room-specific subsamples in several rounds, the repeated dataset was unbalanced and was reduced to 112 home-round observations for mixed-effects analysis (Section 2.6 and Table S5). Dust was swept using pre-cleaned brooms, wrapped in aluminum foil, transported to the laboratory, sieved through a 500 μm stainless-steel mesh, and homogenized. Sample extraction and LC-MS/MS determination were completed in 2016 immediately after the corresponding collection rounds; samples were held at −20 °C only during the short interval between collection and analysis.

The archived household questionnaire retained refurbishment timing and counts of selected furnishings and electrical/electronic products. Building age, dwelling type, floor level, occupancy, ventilation behavior, cleaning or vacuuming frequency, exact time since last cleaning, socioeconomic indicators, detailed geographic distribution, and weather conditions immediately before sampling were not recorded systematically. These unmeasured variables limit representativeness and causal interpretation of seasonal patterns.

For an exploratory comparison among indoor microenvironments, composite office dust was collected monthly at Tongji University from July to December 2016 except November (*N* = 5), and dormitory dust was collected monthly from September to December 2016 (*N* = 4). These limited samples were used descriptively for concentration and congener-profile comparisons; they were not used to infer complete seasonal patterns.

### 2.3. Extraction and Cleanup

Approximately 150 mg of homogenized dust was weighed into a glass centrifuge tube and fortified with isotope-labelled standards. Samples were extracted with 20 mL n-hexane/acetone (1:1, *v*/*v*), vortexed for 1 min, ultrasonicated for 20 min, and centrifuged at 3000 rpm for 10 min. The extraction was repeated twice, and the combined supernatants were concentrated to approximately 1 mL under a gentle nitrogen stream. Solid-phase extraction (SPE) cleanup was performed using Florisil cartridges conditioned with 5 mL methanol followed by 5 mL n-hexane. After sample loading, the tube was rinsed with 3 mL n-hexane and matrix interferences were removed with another 3 mL n-hexane. Target compounds were eluted with 8 mL ethyl acetate, evaporated to near dryness under nitrogen, and reconstituted in 1 mL methanol.

### 2.4. Instrumental Analysis

OPFRs were quantified by liquid chromatography–tandem mass spectrometry (LC-MS/MS; TSQ Quantum Access Max, Thermo Fisher Scientific, Waltham, MA, USA). Separation was achieved using a Hypersil Gold C8 column (50 × 2.1 mm) at 45 °C. The mobile phases were ultrapure water containing 0.1% formic acid (A) and acetonitrile containing 0.1% formic acid (B), delivered at 0.30 mL·min^−1^. The gradient was 55% A at 0–2 min, linearly decreased to 10% A at 8 min, held to 9 min, and returned to 55% A at 11 min. The injection volume was 10 μL. Positive electrospray ionization was used with a spray voltage of 4000 V, capillary temperature of 300 °C, collision-gas pressure of 1.5 mTorr, and multiple-reaction monitoring. Compound-specific transitions are listed in Table S2.

### 2.5. Quality Assurance and Quality Control

Glassware was baked at 400 °C for 5 h, cooled under aluminum foil, and rinsed with methanol before use. Each analytical batch contained three procedural blanks and two samples of NIST Standard Reference Material (SRM) 2585, Organic Contaminants in House Dust (National Institute of Standards and Technology, Gaithersburg, MD, USA). Eleven-point calibration curves ranged from 0.1 to 400 μg·L^−1^ and had coefficients of determination above 0.99. Because TCEP was consistently present in procedural blanks, study-specific operational thresholds were defined as the mean blank concentration plus three standard deviations (detection) and plus five standard deviations (quantification). The latter is explicitly identified as an operational blank-based LOQ rather than a conventional 10-SD LOQ. For other analytes, signal-to-noise ratios of 3 and 10 were used. Method detection limits were 3.7–21 ng·g^−1^. A 25 μg·L^−1^ mixed standard was analyzed after every 10 samples. Mean recoveries were 77.3–125.3% in fortified blanks and 69.2–129.1% in fortified matrices. The archived QA/QC summary retained these ranges but not a reliable analyte-specific attribution of the two extreme matrix values; therefore, no compound identity is assigned retrospectively. Concentrations were not recovery-corrected; isotope-labelled standards were used throughout quantification. SRM results were comparable with published indicative values [20,21] (Tables S3 and S4).

### 2.6. Statistical Analysis

Statistical analyses were performed using SPSS version 23. Concentrations below the method detection limit were replaced with one-half of the compound-specific method detection limit, whereas values between the detection and quantification limits were replaced with LOQ/√2. This simple substitution was retained to reproduce the original 2016 analysis and is reported transparently because substitution can influence estimates for sparsely detected analytes [22]. Concentrations were log_10_-transformed before repeated-measures modelling. One residence contributed multiple room-specific samples within several collection rounds; compound concentrations were combined within each home and sampling month using the geometric mean. This produced 112 home-round observations from 26 residences (Table S5).

Seasonal differences were evaluated using linear mixed-effects models with season as a fixed effect and residence identity as a random intercept, thereby accounting for the unbalanced repeated sampling. Residual-versus-fitted and normal Q–Q plots of the log_10_-scale residuals were reviewed and did not indicate severe heteroscedasticity or departure from normality. A random-intercept-only structure was retained because winter and summer were each represented by one sampling month, spring and autumn by two, and many homes lacked one or more rounds; random seasonal slopes could not be estimated robustly from this sparse, unbalanced design. Season-specific numbers of observations and residences are reported in Table S5. Model-estimated marginal means were back-transformed and reported as geometric means with 95% confidence intervals. Six pairwise seasonal contrasts were adjusted by the Holm method. TPrP was excluded because its residential detection frequency was 2.21%. Spearman correlations were used for congener co-occurrence, and Pearson correlation for the TBOEP product-count association. Statistical significance was defined as Holm-adjusted *p* < 0.05. Repeated-measures exposure data require explicit consideration of within-subject clustering [23].

### 2.7. Exposure and Screening-Level Risk Assessment

A retrospective exposure scenario corresponding to the measured 2016 dust concentrations was estimated for infants aged 1–3 years and adults aged ≥18 years. Uptake-adjusted estimated daily intakes (EDIs) were calculated separately for dust ingestion, inhalation, and dermal absorption. One compound-specific factor was applied per pathway: gastrointestinal bioaccessibility for ingestion, pulmonary bioaccessibility for inhalation, and an absorption fraction for dermal contact. Pathway estimates were summed only to describe their relative contributions; the sum was not compared directly with an oral toxicity benchmark. Mean and 95th-percentile dust concentrations represented the mean-concentration and 95th-percentile-concentration scenarios, respectively. Parameters were drawn from published sources [24,25,26,27,28,29,30,31,32,33,34]. Equations and details are provided in Text S1 and Table S8.

For screening against oral reference doses, an oral administered dose was calculated from dust concentration, ingestion rate, indoor residence fraction, and body weight without gastrointestinal bioaccessibility or oral bioavailability adjustment. The ingestion hazard quotient (HQ_ing_) was this administered dose divided by the corresponding oral reference dose; HQ_ing_ > 1 was considered a screening-level indication of potential concern [35,36]. Inhalation and dermal estimates were not converted to oral-equivalent doses because route-specific toxicity benchmarks and validated route-to-route extrapolation factors were unavailable. Oral cancer slope factors of 2 × 10^−2^ and 9 × 10^−3^ (mg·kg^−1^·day^−1^)^−1^ were used for TCEP and TBP, respectively; the TBP value is the U.S. EPA PPRTV provisional oral slope factor [12,37]. TDCIPP was assessed using the California no-significant-risk-level approach [38]. These calculations indicate benchmark exceedance only under the modelled 2016 oral-ingestion scenarios and do not account for mixture toxicity, toxicity-value uncertainty, or unmeasured replacement OPFRs.

## 3. Results

### 3.1. Occurrence and Concentrations in Indoor Dust

At least one target OPFR was detected in every sample. In residential dust, the median and mean Σ_8_OPFR concentrations were 1.51 × 10^3^ and 4.35 × 10^3^ ng·g^−1^, respectively; the range was 86.2–5.07 × 10^4^ ng·g^−1^ (Table 1). Corresponding median (mean) values were 2.48 × 10^3^ (3.36 × 10^3^) ng·g^−1^ in the five office composites and 673 (963) ng·g^−1^ in the four dormitory composites. The mean-minus-median difference in residences was 2.84 × 10^3^ ng·g^−1^, consistent with a right-skewed distribution. Maximum residential concentrations of TCEP and TBOEP were 2.70 × 10^4^ and 1.00 × 10^4^ ng·g^−1^, respectively. Because the three microenvironments differed in sampling months, sample numbers, and compositing design, these descriptive values are not interpreted as a concentration ranking.

**Figure 1:**
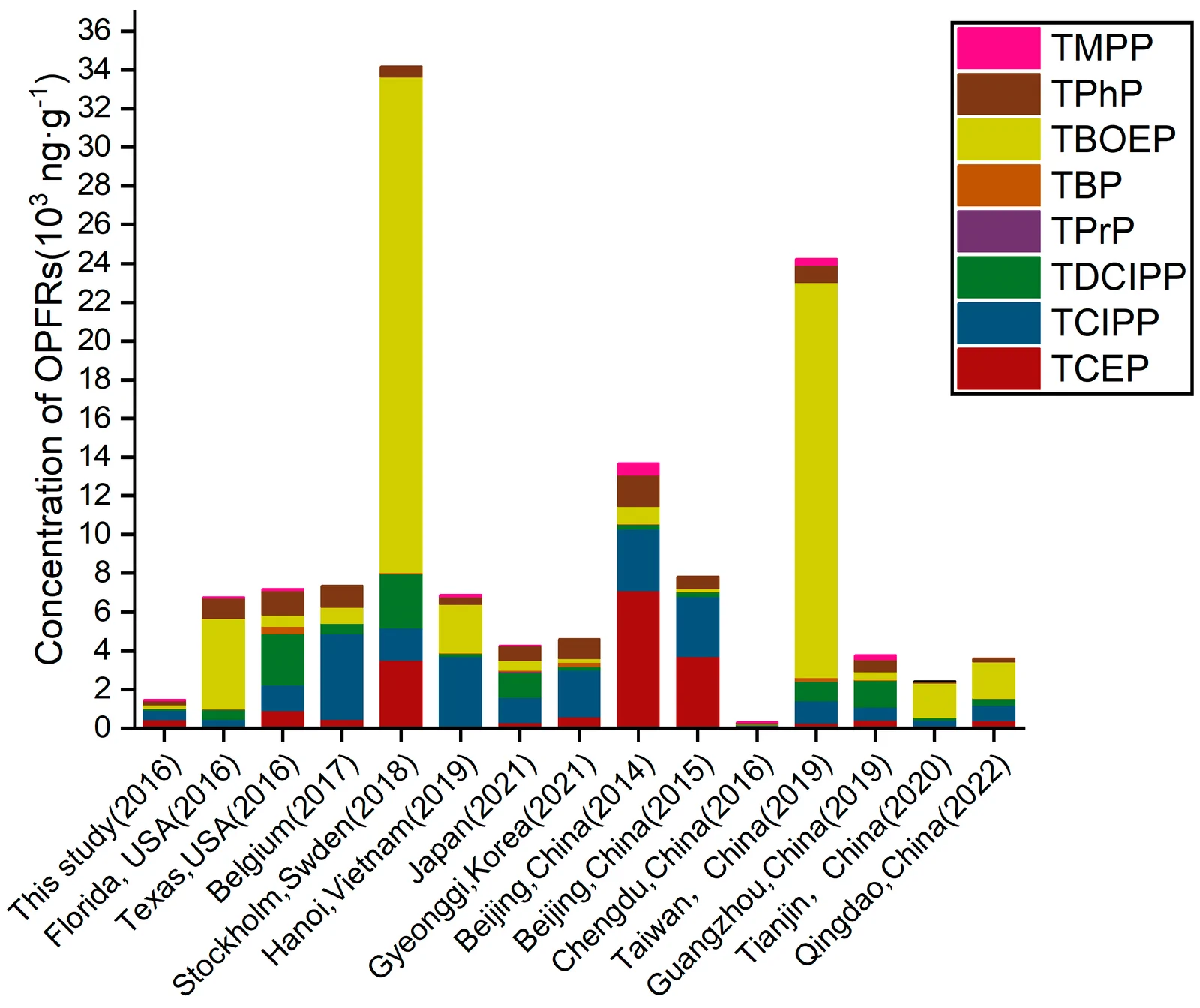
Median concentrations and congener compositions of OPFRs in indoor dust from the present residential dataset and selected studies. All plotted concentrations are medians expressed as 10^3^ ng·g^−1^. Years in parentheses indicate sampling years.

The median residential Σ_8_OPFR concentration was within the broad range reported internationally (Figure 1). It was lower than values reported for several U.S., European, Beijing, Stockholm, and Taiwan datasets, but comparable to surveys from Tianjin, Guangzhou, Qingdao, and Japan [39,40,41,42,43,44,45,46,47,48,49]. Cross-study comparisons remain descriptive because analytical target lists, particle-size fractions, collection methods, building types, and sampling years differ.

### 3.2. Congener Profiles and Exploratory Microenvironmental Differences

**Figure 2:**
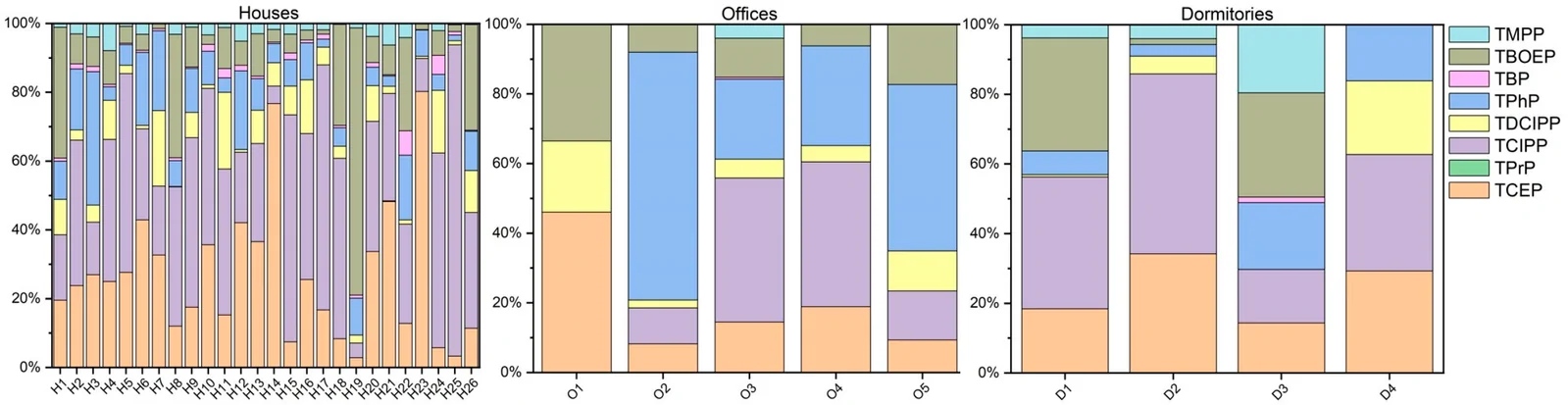
Relative composition profiles of eight OPFRs in dust from residences (H1–H26), offices (O1–O5), and dormitories (D1–D4). Office and dormitory results are exploratory because of the limited sample numbers.

Chlorinated OPFRs were the dominant chemical class in all three microenvironments. In residential dust, the mean within-sample fractions of TCIPP and TCEP were 35.2% and 29.0% of Σ_8_OPFRs, respectively. These percentages are averages of the fraction calculated within each sample (mean of ratios) and therefore need not equal the ratios calculated from the arithmetic means in Table 1. Residential profiles were heterogeneous: TBOEP accounted for 77.7% of the total at H19, whereas TPhP accounted for 38.8% at H3 (Figure 2). Office composites had larger mean fractions of TPhP, TCIPP, and TCEP, whereas dormitory composites were generally dominated by TCIPP and TCEP with notable TBOEP or TPhP contributions in individual samples. These are descriptive profile contrasts only.

**Figure 3:**
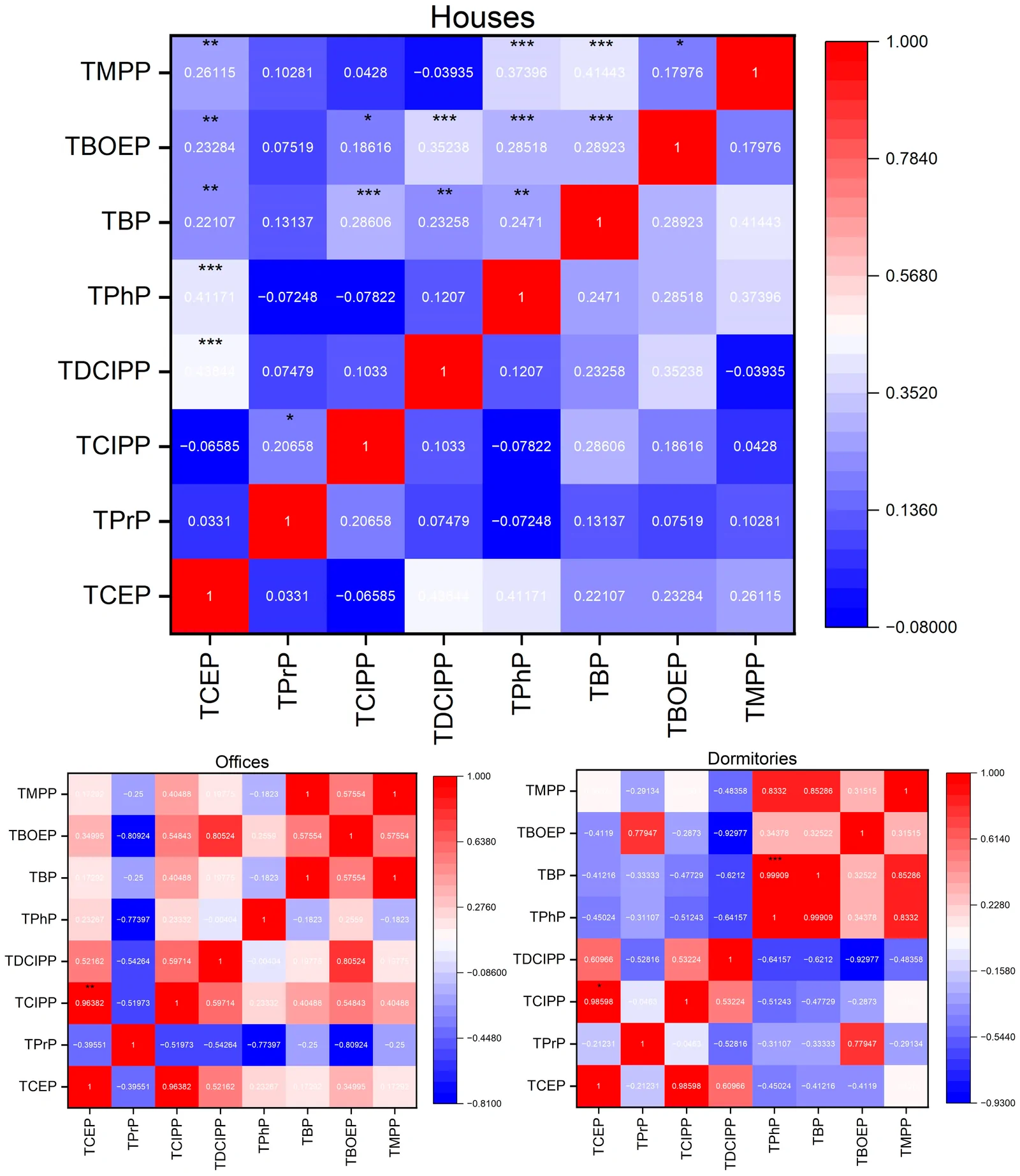
Spearman correlation matrices for OPFR congeners in residential, office, and dormitory dust. Values are Spearman ρ coefficients. *, **, and *** indicate *p* < 0.05, *p* < 0.01, and *p* < 0.001, respectively. Office and dormitory panels are exploratory because of the small sample sizes.

The residential Spearman correlation matrix showed positive correlations of TCEP with TCIPP (ρ = 0.55), TDCIPP (ρ = 0.44), TPhP (ρ = 0.41), TBOEP (ρ = 0.23), and TMPP (ρ = 0.26) (Figure 3). TPhP was positively correlated with TBP, TBOEP, and TMPP, and TDCIPP with TBOEP and TBP. Strong TCEP–TCIPP coefficients were also observed in the office and dormitory composites, but estimates based on *N* = 5 or *N* = 4 are unstable.

### 3.3. Household Characteristics and Potential Source Indicators

Residential samples were grouped according to refurbishment timing. Median concentrations of TCEP, TCIPP, TDCIPP, TPhP, and TMPP were higher in homes refurbished after 2015 than in homes refurbished earlier, although only TBP differed significantly (*p* = 0.0017; Figure S1).

TBOEP concentrations were positively associated with the combined number of polyurethane-foam-containing furnishings and electrical/electronic products (Pearson r = 0.57, *p* = 0.0014; Figure S2).

### 3.4. Seasonal Variability in Residential Dust

Formal seasonal comparisons were restricted to the residential dataset because only residences were sampled across all four seasons. After aggregation of room-specific subsamples within home-month and adjustment for repeated measurements, season had a significant overall effect on Σ_8_OPFRs (global *p* < 0.001) and on every frequently detected congener included in the mixed-effects analysis (global *p* = 0.024 for TBOEP to *p* < 0.001 for TCEP, TDCIPP, TBP, TMPP, and Σ_8_OPFRs; Tables S6 and S7). The model-estimated geometric mean of Σ_8_OPFRs was 3044 ng·g^−1^ in spring, 2366 ng·g^−1^ in summer, 1555 ng·g^−1^ in autumn, and 3319 ng·g^−1^ in winter. Spring and winter concentrations were 1.96-fold (Holm-adjusted *p* < 0.001) and 2.13-fold (*p* < 0.001) higher than autumn, respectively.

**Figure 4:**
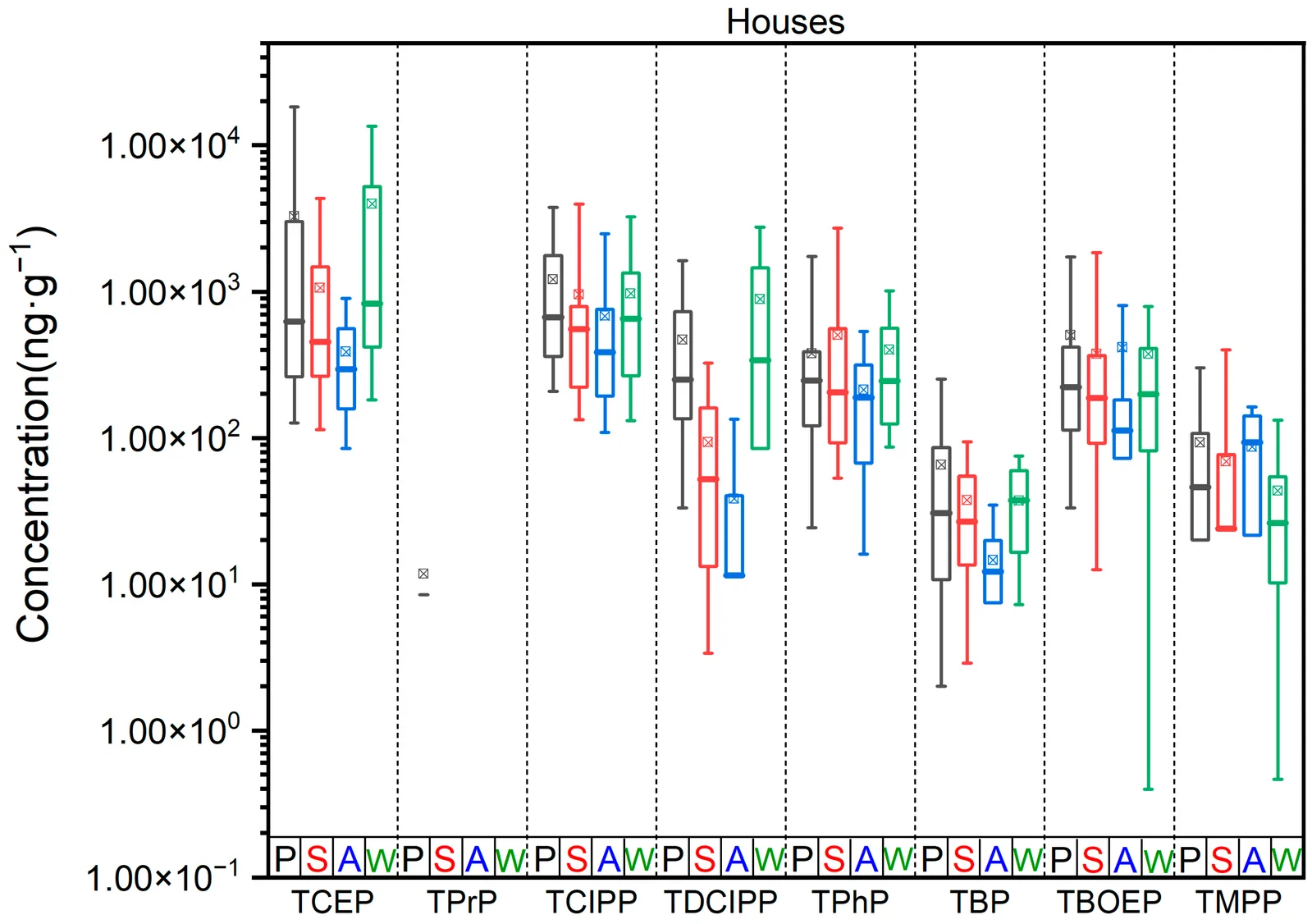
Seasonal distributions of OPFR concentrations in residential dust collected during 2016. P, spring; S, summer; A, autumn; W, winter. Boxes show medians and interquartile ranges.

Congener-specific contrasts supported the same general pattern while revealing chemical-specific behavior (Figure 4). Relative to autumn, TCEP was 2.31-fold higher in spring and 3.21-fold higher in winter (both adjusted *p* < 0.001), TCIPP was 1.62-fold higher in spring (*p* = 0.007), and TBOEP was 1.83-fold higher in spring (*p* = 0.029). TDCIPP showed the strongest seasonality. TBP was lower in autumn than spring, summer, and winter. In contrast, TMPP was 5.19-fold higher in spring than summer and 4.18-fold higher in autumn than summer. Because 14.7% of residential TMPP measurements were below detection and substituted, the reversed TMPP pattern should be interpreted cautiously; an influence of month-specific imputation cannot be excluded. TPrP was not modelled because of its 2.21% detection frequency. Here and in Tables S6 and S7, “winter” and “summer” refer specifically to the January and July sampling windows, respectively, rather than averages across the full seasons. Therefore, contrasts involving winter or summer should be interpreted as differences among the sampled seasonal windows and not as estimates generalizable to all winter or summer months.

### 3.5. Retrospective Exposure and Risk Estimates

Under the mean 2016 concentration scenario, uptake-adjusted total Σ_8_OPFR EDIs were 3.3 ng·kg^−1^·day^−1^ for infants and 0.65 ng·kg^−1^·day^−1^ for adults (Table 2). Under the 95th-percentile-concentration scenario, corresponding totals were 44 and 8.6 ng·kg^−1^·day^−1^. Dust ingestion contributed 96.0% and 95.6% of infant totals in the mean and 95th-percentile-concentration scenarios, respectively. In adults, ingestion contributed 67.5% in both scenarios and dermal absorption contributed 32.2% and 30.3%, respectively (Figure S4). Inhalation made the smallest contribution. These pathway sums describe uptake-adjusted exposure and were not used as the numerator for oral benchmark comparison.

TCEP and TCIPP were the largest contributors to uptake-adjusted intake. Mean total EDIs for infants were 1.2 ng·kg^−1^·day^−1^ for TCEP and 1.3 ng·kg^−1^·day^−1^ for TCIPP, increasing to 29 and 8.6 ng·kg^−1^·day^−1^ under the 95th-percentile-concentration scenario. Corresponding adult means were 0.23 and 0.24 ng·kg^−1^·day^−1^. Oral-ingestion HQ values calculated on an administered-dose basis were below 1 for all evaluated compounds (Figure S5). Oral carcinogenic screening values for TCEP, TBP, and TDCIPP were also below the selected 10^−6^–10^−4^ screening range under the modelled scenarios (Figure S6).

## 4. Discussion

### 4.1. Historical Context and Microenvironmental Heterogeneity

The principal value of this dataset is its explicit temporal context and year-round residential coverage. The measurements document OPFR contamination in Shanghai indoor dust during 2016, a period in which OPFR production and use were already substantial but product formulations and regulatory attention continued to evolve [3,4]. The complete dataset is being reported now because it provides a pre-2020 benchmark analyzed immediately after collection and re-evaluated with a repeated-measures model. It should not be used to represent current Shanghai concentrations or to infer a decade-scale trend without method-matched contemporary sampling.

The dominance of chlorinated OPFRs is consistent with Chinese and international dust surveys [5,6,7,8,9]. The broad residential range and household-specific profiles indicate substantial source heterogeneity. However, the convenience panel was not designed to represent the socioeconomic, geographic, or building-stock distribution of Shanghai. Moreover, office and dormitory collections covered different months, used composite samples, and were very small. Their distributions therefore generate hypotheses only and do not establish a house–office–dormitory ranking.

### 4.2. Source-Related Interpretation

The higher median concentrations in recently refurbished homes and the TBOEP product-count association are compatible with known OPFR applications in polyurethane foam, electronics, building materials, floor treatments, and coatings [50,51,52,53,54]. Direct measurements of Chinese building materials show that products can contain and emit multiple OPFRs [50]. Nevertheless, the Results report only the observed group contrasts and correlation. Source interpretation remains in this discussion because settled-dust concentrations reflect source strength together with sorption, migration, deposition, resuspension, and removal [54]. The questionnaire did not identify product-specific emissions, so these associations cannot establish causality. Future work should combine product screening, chamber-emission measurements, and receptor or source-apportionment models.

### 4.3. Interpretation of Seasonal Variability

The lower autumn and higher spring/winter concentrations of several chlorinated congeners agree with repeated monitoring of floor dust from three offices in Beijing, China, where phosphorus flame retardants were generally higher in colder seasons [17]. Residential work in Taiwan and microenvironmental studies in Qingdao also reported seasonal variation, although direction and magnitude differed among compounds, media, and sites [15,16]. Temporal studies of semivolatile organic compounds indicate that source inventories, ventilation, cleaning, and occupant activities can contribute to within-building variation [18,19].

Dust and air need not show parallel seasonal patterns. Prior studies suggest that warmer conditions can increase emissions to air, whereas low air exchange may favor retention in settled dust [15,16,17,18,19]. Ventilation, cleaning, resuspension, and occupant behavior are therefore plausible explanations, not variables demonstrated by this dataset. Because temperature, humidity, weather before sampling, air-exchange rate, and cleaning timing were not recorded, the mixed-effects results establish statistical seasonal differences only; they do not identify the responsible mechanism. Nevertheless, the mixed-effects results demonstrate that the principal autumn minima persisted after accounting for repeated observations within residences, strengthening the evidence that the pattern was not solely caused by differences in the homes sampled in each season. Additionally, the one-month winter and summer windows also prevent separation of a true season effect from January- or July-specific variability.

More importantly, winter and summer were represented only by January and July, respectively. Their mixed-model marginal means should therefore be regarded as month-specific snapshots rather than averages across the full winter and summer seasons. An atypical temperature, ventilation pattern, cleaning event, occupancy pattern, or antecedent weather condition in either sampled month could shift the corresponding estimate upward or downward, thereby exaggerating or attenuating pairwise seasonal contrasts. By comparison, spring and autumn each included two months and may average some within-season variability, providing somewhat broader temporal coverage for those estimates. Although the mixed-effects model accounts for repeated measurements and missing residence-level sampling rounds, it cannot estimate a separate month-within-season effect because winter and summer have no within-season monthly replication. We therefore interpret contrasts involving winter or summer as differences among the sampled seasonal windows rather than effects generalizable to every winter or summer month.

### 4.4. Exposure Implications

The higher uptake-adjusted EDI for infants reflects age-specific dust ingestion and body weight assumptions. The resulting pathway totals are useful for comparing relative contributions, but they mix bioaccessible ingestion and inhalation estimates with absorbed dermal estimates and are not toxicological equivalents. Accordingly, only the unadjusted oral administered dose was compared with oral RfDs and oral cancer slope factors. No route-to-route extrapolation was made for inhalation or dermal contact. Ingestion dominated infant exposure, while dermal absorption was nearly as important as ingestion in adults. Similar age-dependent contrasts have been reported elsewhere, although the dominant pathway varies substantially with model assumptions and bioaccessibility parameters [24,25,26,27,35,43]. For example, much higher estimates and a larger dermal contribution were reported in Hanoi [43]. Such differences demonstrate that EDI, HQ, and cancer-risk values are model-dependent screening tools rather than direct measures of absorbed dose.

The screening calculations indicate no exceedance of the selected oral benchmarks under the modelled 2016 mean- or 95th-percentile-concentration scenarios; they do not demonstrate an absence of health risk. The assessment used deterministic point estimates, considered compounds separately, did not quantify mixture effects or replacement OPFRs, and relied on toxicity values with different evidentiary status, including a provisional TBP slope factor. Inhalation and dermal risks were not benchmarked because route-specific values were unavailable. Contemporary biomonitoring and probabilistic mixture-based assessment are needed to characterize current risk.

### 4.5. Strengths and Limitations

Strengths include the large residential sample set, four-season repeated collection, completion of chemical analysis immediately after collection in 2016, extensive analytical quality control using SRM 2585, evaluation of household descriptors, and multi-pathway exposure assessment. Reanalysis of the original residential data using mixed-effects models also accounted for within-home correlation and the unbalanced sampling structure. The study therefore preserves a dated, seasonally resolved baseline that can be used for future method-matched comparison.

Several limitations require emphasis. First, samples were collected and analyzed in 2016 and do not represent present-day exposure; no method-matched contemporary samples were available. Second, residences formed a small convenience panel, and building, occupancy, ventilation, cleaning, socioeconomic, detailed geographic, and pre-sampling weather data were not systematically recorded. Third, the temporal coverage was asymmetric: winter and summer were represented only by January and July, respectively, whereas spring and autumn each combined two months. Month is therefore inseparable from season for the winter and summer categories. The winter and summer marginal means, together with their confidence intervals, are conditional on the sampled January and July windows and do not capture variability among other winter or summer months. If January or July was atypical, the corresponding seasonal contrasts may have been exaggerated or attenuated. The sparse design supported a random-intercept model but did not permit estimation of a separate month-within-season effect or robust random seasonal slopes. Fourth, office and dormitory samples were few, composite, and incompletely seasonal. Fifth, substitution for censored values may have influenced the TMPP summer estimate. Sixth, only eight conventional OPFRs were measured. Finally, pathway EDIs depend on deterministic exposure and bioaccessibility assumptions, and oral screening did not quantify inhalation, dermal, mixture, or toxicity-value uncertainty. These limitations guide interpretation and follow-up design.

## 5. Conclusions

This study establishes a seasonally resolved 2016 historical reference for OPFRs in Shanghai indoor dust. OPFRs were ubiquitous, chlorinated congeners dominated, and residential dust showed substantial household heterogeneity. Office and dormitory results were exploratory and insufficient for a microenvironmental ranking. Mixed-effects models confirmed lower autumn concentrations for Σ_8_OPFRs and several major congeners, while TMPP followed a distinct but censoring-sensitive profile. Refurbishment timing and product inventories provided source-related clues. Under the modelled 2016 scenario, uptake-adjusted intake was higher for infants, and oral administered-dose screening values did not exceed the selected benchmarks. Method-matched resampling, expanded target lists, biomonitoring, and probabilistic mixture assessment are needed to determine how exposure has changed since 2016.

## Figures and Tables

**Table 1:** Concentrations (ng·g^−1^) of OPFRs in residential, office, and dormitory dust.

	TCEP	TPrP	TCIPP	TDCIPP	TPhP	TBP	TBOEP	TMPP	Alkyl- OPFRs	Aryl-OPFRs	Cl- OPFRs	Σ_8_ OPFRs
Residences (*N* = 136 raw samples from 26 homes)												
D.F.	100%	2.21%	100%	79.4%	97.1%	89.0%	94.1%	85.3%	100%	100%	100%	100%
Min	42.6	N.D.	43.6	N.D.	N.D.	N.D.	N.D.	N.D.	N.D.	N.D.	86.2	86.2
Median	447	N.D.	522	104	212	20.4	163	44.5	183	256	1.07 × 10^3^	1.51 × 10^3^
Max	2.70 × 10^4^	321	5.49 × 10^3^	3.31 × 10^3^	3.24 × 10^3^	367	1.00 × 10^4^	866	1.07 × 10^4^	4.11 × 10^3^	3.58 × 10^4^	5.07 × 10^4^
Mean	2.14 × 10^3^	4.50	965	342	347	40.8	433	79.2	478	426	3.44 × 10^3^	4.35 × 10^3^
Offices (*N* = 5 composite samples)												
D.F.	100%	0.00%	80.0%	100%	80.0%	20.0%	100%	20.0%	100%	80.0%	100%	100%
Min	228	N.D.	N.D.	82.0	N.D.	N.D.	166	N.D.	166	N.D.	310	494
Median	290	N.D.	366	232	1.22 × 10^3^	N.D.	371	N.D.	371	1.22 × 10^3^	888	2.48 × 10^3^
Max	1.12 × 10^3^	N.D.	2.47 × 10^3^	293	2.52 × 10^3^	24.9	479	172	504	2.69 × 10^3^	3.89 × 10^3^	7.09 × 10^3^
Mean	499	N.D.	994	197	1.29 × 10^3^	4.97	349	34.4	354	1.32 × 10^3^	1.69 × 10^3^	3.36 × 10^3^
Dormitories (*N* = 4 composite samples)												
D.F.	100%	0.00%	100%	75.0%	100%	25.0%	75.0%	75.0%	75.0%	100%	100%	100%
Min	99.1	N.D.	107	N.D.	60.8	N.D.	N.D.	N.D.	N.D.	60.8	206	267
Median	145	N.D.	245	45.3	64.0	N.D.	119	55.1	119	119	435	673
Max	623	N.D.	940	93.1	132	11.1	306	136	317	268	1.66 × 10^3^	2.24 × 10^3^
Mean	253	N.D.	384	45.9	80.3	2.76	136	61.4	139	142	683	963

Alkyl-OPFRs represent the sum of TPrP, TBP, and TBOEP. Aryl-OPFRs represent the sum of TPhP and TMPP. Chlorinated OPFRs represent the sum of TCEP, TCIPP, and TDCIPP. D.F., detection frequency; N.D., not detected.

**Table 2:** Uptake-adjusted EDIs of OPFRs via dust ingestion, dust inhalation, and dermal absorption for infants and adults.

Analyte	Dust Ingestion (ng·kg^−1^·day^−1^)		Dust Inhalation (ng·kg^−1^·day^−1^)		Dermal Absorption (ng·kg^−1^·day^−1^)		Total Intake (ng·kg^−1^·day^−1^)	
	Mean	95th Percentile	Mean	95th Percentile	Mean	95th Percentile	Mean	95th Percentile
Infants								
TCEP	1.2	28	8.0 × 10^−4^	0.20	0.039	0.93	1.2	29
TCIPP	1.2	8.2	9.3 × 10^−4^	0.065	0.045	0.30	1.3	8.6
TDCIPP	0.12	2.0	1.0 × 10^−4^	0.018	0.0087	0.15	0.13	2.2
TPrP	Excl.	Excl.	Excl.	Excl.	Excl.	Excl.	Excl.	Excl.
TBP	0.038	0.30	3.5 × 10^−5^	0.0029	0.0016	0.013	0.040	0.32
TBOEP	0.38	2.4	3.2 × 10^−4^	0.020	0.015	0.091	0.40	2.5
TPhP	0.20	1.2	1.0 × 10^−4^	0.0066	0.019	0.11	0.22	1.3
TMPP	0.028	0.14	2.0 × 10^−6^	1.0 × 10^−4^	0.0040	0.019	0.032	0.16
ΣOPFRs	3.2	42	0.0023	0.32	0.13	1.6	3.3	44
Adults								
TCEP	0.16	3.8	4.9 × 10^−4^	0.12	0.063	1.5	0.23	5.4
TCIPP	0.17	1.1	5.7 × 10^−4^	0.040	0.073	0.49	0.24	1.7
TDCIPP	0.017	0.28	6.3 × 10^−5^	0.011	0.014	0.24	0.031	0.53
TPrP	Excl.	Excl.	Excl.	Excl.	Excl.	Excl.	Excl.	Excl.
TBP	0.0052	0.040	2.2 × 10^−5^	0.0017	0.0027	0.021	0.0079	0.063
TBOEP	0.054	0.32	1.9 × 10^−4^	0.013	0.024	0.15	0.078	0.48
TPhP	0.028	0.17	6.3 × 10^−5^	0.0040	0.030	0.18	0.058	0.35
TMPP	0.0040	0.019	1.2 × 10^−6^	6.1 × 10^−5^	0.0065	0.032	0.011	0.052
ΣOPFRs	0.44	5.8	0.0014	0.19	0.21	2.6	0.65	8.6

Excl., excluded from calculation because the residential detection frequency was 2.21%.

## Data Availability

Data are included in this article or Supplementary Materials.

## Supplementary Materials

The following supporting information can be downloaded at: https://www.mdpi.com/article/10.3390/toxics14090758/s1, Table S1: Chemical properties of target OPFR compounds [55]; Table S2: Multiple-reaction-monitoring conditions and method detection limits for OPFR analysis; Table S3: Instrument performance, limits of detection, and limits of quantification; Table S4: OPFR concentrations in SRM 2585 house-dust standard reference material; Table S5: Distribution of residential dust samples and home-round observations used in the repeated-measures analysis; Table S6: Estimated marginal geometric means (95% confidence intervals) from linear mixed-effects models of residential OPFR concentrations (ng·g^−1^); Table S7: Pairwise seasonal concentration ratios and Holm-adjusted p-values from the linear mixed-effects models; Table S8: Key parameters used for retrospective exposure assessment; Text S1: Detailed Exposure and Health-Risk Assessment; Figure S1: Comparison of OPFR concentrations in dust between newly refurbished homes (refurbished after 2015) and earlier-refurbished homes. ** *p* < 0.01; Figure S2: Pearson correlation between TBOEP concentrations in residential dust and the combined number of polyurethane-foam-containing furnishings and electrical/electronic products; Figure S3: Seasonal trends in median concentrations of OPFR congeners in the year-round residential dust dataset; Figure S4: Contributions of ingestion, inhalation, and dermal absorption to uptake-adjusted total EDI in infants and adults under the mean- and 95th-percentile-concentration scenarios; Figure S5: Oral-ingestion hazard quotients calculated from administered dust-ingestion doses for infants and adults under the mean- and 95th-percentile-concentration scenarios. TPrP was excluded because its residential detection frequency was 2.21%; Figure S6: Oral carcinogenic screening values for TCEP, TBP, and TDCIPP in infants and adults under the 95th-percentile-concentration scenario. Dashed lines indicate the 10^−6^ and 10^−4^ screening levels.
