# Supplementary material for: Organophosphate Flame Retardants in Shanghai Indoor Dust: A 2016 Historical Baseline of Seasonal Variability, Microenvironmental Profiles, and Human Exposure
Source: Toxics. 2026 Aug 26;14(9):758. doi: 10.3390/toxics14090758 (PMC13611237; doi:10.3390/toxics14090758)
Supplement: Supplementary file 1 [file toxics-14-00758-s001.zip › toxics-4445615-supplementary.pdf]

Supporting information for

Organophosphate Flame Retardants in Shanghai Indoor Dust: A 2016 Historical Baseline of Seasonal Variability, Microenvironmental Profiles, and Human Exposure

Jiajun Chang [1,2]†, Li Li [1,2]†, Binghong Cheng [3], Zhiliang Zhu [1,2], Qinghui Huang [1,2], Yanling Qiu [1,2],* and Åke Bergman [4]

[1] Key Laboratory of Yangtze River Water Environment, College of Environmental Science and Engineering, Tongji University, 1239 Siping Road, Shanghai 200092, China

[2] Shanghai Institute of Pollution Control and Ecological Security, 1239 Siping Road, Shanghai 200092, China

[3] Hangzhou Ecological Environment Publicity and Education Information Center, Hangzhou 310020, China

[4] Department of Environmental Science and Analytical Chemistry, Stockholm University, 10691 Stockholm, Sweden

† These authors contributed equally to this work.

* Correspondence: ylqiu@tongji.edu.cn; Tel.: +86-13916924371

Table S1. Chemical properties of target OPFR compounds [55].

Compounds	Abbr.	CAS No.	MW	Formula	S	VP	H	$logK_{OW}$	$logK_{OC}$	$logK_{OA}$	BCF
Tripropyl phosphate	TPrP	513-08-6	224.23	$C_9H_{21}O_4P$	6.45E+3	2.31E-2	1.36E-6	2.35	2.47	6.42	1.20
Tri-n-butyl phosphate	TBP	126-73-8	266.31	$C_{12}H_{27}O_4P$	7.35	3.49E-3	3.19E-6	3.82	3.27	9.21	30.2
Tris(2-butoxyethyl) phosphate	TBOEP	78-51-3	398.47	$C_{18}H_{39}O_7P$	1.10E+3	1.23E-6	1.20E-11	3.00	5.66	13.0	1.08E+3
Tris(2-chloroethyl) phosphate	TCEP	115-96-8	285.49	$C_6H_{12}C_{l3}O_4P$	7.00E+3	3.91E-4	2.55E-8	1.63	2.47	7.42	1.37
Tris(2-chloroisopropyl) phosphate	TCIPP	13674-84-5	327.57	$C_9H_{18}C_{l3}O_4P$	1.20E+3	5.64E-5	5.96E-8	2.89	3.10	8.20	3.55
Tris(1,3-dichloro-2-propyl) phosphate	TDCIPP	13674-87-8	430.90	$C_9H_{15}Cl_6O_4P$	7.00	2.86E-7	2.61E-9	3.65	3.96	10.6	13.5
Triphenyl phosphate	TPhP	115-86-6	326.28	$C_{18}H_{15}O_4P$	1.90	4.72E-7	3.98E-8	4.70	3.71	8.45	113
Tris(methylphenyl) phosphate (tricresyl phosphate; isomer mixture)	TMPP	1330-78-5	368.36	$C_{21}H_{21}O_4P$	0.300	3.49E-8	5.35E-8	6.34	4.34	12.0	164

MW, molecular weight ($g \cdot mol^{-1}$); S, water solubility at 25 °C ($mg \cdot L^{-1}$); VP, vapor pressure at 25 °C (mmHg); H, Henry's law constant at 25 °C ($atm \cdot m^3 \cdot mol^{-1}$); K_{OW}, octanol–water partition coefficient; K_{OC}, organic carbon–water partition coefficient; K_{OA}, octanol–air partition coefficient; BCF, bioconcentration factor ($L \cdot kg^{-1}$ wet weight). Data were compiled from van der Veen and de Boer [1] and, where applicable, estimated using the U.S. EPA EPI Suite^{TM} v4.11 [55].

Table S2. Multiple-reaction-monitoring conditions and method detection limits for OPFR analysis.

Compounds	Internal Standard	Quantitative transition (amu)	Collision energy (eV)	MDLs (ng·g^{-1})
TCEP	TCEP-d12	284.8-98.9	27	21
		284.8-160.7	16	
TCIPP	TCEP-d12	326.8-98.9	29	6.7
		326.8.174.6	13	
TDCIPP	TDCIPP-d15	430.5-98.8	32	16
		430.5-208.5	16	
TPrP	TBP-d27	224.8-140.9	15	5.3
		224.8-98.9	10	
TBP	TBP-d27	266.9-98.9	19	4.9
		266.9-154.8	10	
TBOEP	TBP-d27	398.9-298.6	12	8.0
		398.9-198.5	15	
TPhP	TPhP-d15	326.9-151.7	34	11
		326.9-214.6	27	
TMPP	TPhP-d15	368.9-164.8	45	3.7
		368.9-242.6	28	
TBP-d27	-	294.1-165.9	12	Na
		294.1-101.9	22	
TCEP-d12	-	297.0-102.0	35	Na
		297.0-82.0	35	
TDCIPP-d15	-	445.5-102.0	30	Na
		445.5-79.0	40	
TPhP-d15	-	341.9-159.5	36	Na
		341.9-221.9	28	

MDLs, method detection limits; Na, not applicable

Table S3. Instrument performance, limits of detection, and limits of quantification.

Compounds	Internal Standard	Retention time (min)	Linearity (R^2) *	LOD (ng·mL^{-1})	LOQ (ng·mL^{-1})
TCEP	TCEP-d12	1.62	0.9923	1.8	3.1
TCIPP	TCEP-d12	3.86	0.9962	0.30	1.0
TDCIPP	TDCIPP-d15	5.85	0.9935	0.74	2.4
TPrP	TBP-d27	2.74	0.9913	0.24	0.80
TBP	TBP-d27	5.96	0.9974	0.22	0.73
TBOEP	TBP-d27	6.54	0.9966	0.36	1.2
TPhP	TPhP-d15	6.01	0.9985	0.50	1.6
TMPP	TPhP-d15	7.52	0.9992	0.16	0.55

Calibration range: 0.1 - 400 ng·mL^{-1}; LOD, limit of detection; LOQ, limit of quantification. The TCEP LOQ is the study-specific operational blank mean + 5 SD threshold. Concentrations were not recovery-corrected.

Table S4. OPFR concentrations in SRM 2585 house-dust standard reference material.

	OPFRs levels in SRM 2585 (mean (SD), $\mu g \cdot g^{-1}$)	OPFRs levels in SRM 2585 (mean (SD), $\mu g \cdot g^{-1}$)	OPFRs levels in SRM 2585 (mean (SD), $\mu g \cdot g^{-1}$)
	This study (n = 4)	Indicative values[a]	Indicative values[b]
TCEP	0.72 (0.11)	0.55 (0.11)	0.79 (0.12)
TCIPP	1.0 (0.020)	1 (0.20)	0.94 (0.26)
TDCIPP	1.9 (0.19)	1.5 (0.24)	1.6 (0.53)
TPrP	N.D.	N.D.	N.D.
TBP	0.27 (0.030)	0.14 (0.020)	0.27 (0.11)
TPhP	0.98 (0.25)	0.87 (0.07)	1.1 (0.099)
TBOEP	49 (22)	63 (15)	73 (32)
TMPP	0.87 (0.16)	0.95 (0.13)	0.84 (0.24)

Values are mean (standard deviation), $\mu g \cdot g^{-1}$. Indicative values were obtained from Brandsma et al. [20] and He et al. [21]. N.D., not detected.

Table S5. Distribution of residential dust samples and home-round observations used in the repeated-measures analysis.

Season	Months represented	Raw dust samples	Unique residences	Home-round observations
Spring	March and May	52	25	44
Summer	July	20	16	16
Autumn	September and November	43	21	35
Winter	January	21	17	17
Total	January, March, May, July, September, and November	136	26	112

Home-round observations represent one value per residence and collection month. When multiple room-specific samples were available within the same home-month, concentrations were combined using the geometric mean. Winter was represented by January; spring by March and May; summer by July; and autumn by September and November.

Table S6. Estimated marginal geometric means (95% confidence intervals) from linear mixed-effects models of residential OPFR concentrations ($ng \cdot g^{-1}$).

Analyte	Global season *p*	Spring	Summer	Autumn	Winter
TCEP	<0.001	584.8 (405.4–843.8)	376.2 (225.3–628.3)	253.5 (170.6–376.9)	813.2 (493.7–1339.4)
TCIPP	0.010	852.9 (607.3–1198.0)	778.7 (506.9–1196.3)	527.2 (368.4–754.5)	746.8 (490.6–1136.8)
TDCIPP	<0.001	204.1 (134.1–310.7)	35.8 (18.6–68.9)	19.9 (12.5–31.7)	163.3 (86.5–308.5)
TPhP	0.015	201.8 (136.0–299.6)	157.5 (89.8–276.2)	114.9 (74.8–176.5)	272.3 (157.5–470.8)
TBP	<0.001	29.5 (20.3–42.8)	20.8 (12.2–35.3)	9.6 (6.4–14.4)	22.2 (13.2–37.1)
TBOEP	0.024	220.6 (127.5–381.6)	200.4 (102.7–390.7)	120.2 (67.8–213.2)	135.8 (70.5–261.8)
TMPP	<0.001	43.7 (27.0–70.5)	8.4 (4.1–17.5)	35.2 (20.7–59.8)	23.9 (11.8–48.7)
Σ_8OPFRs	<0.001	3044.1 (2365.7–3917.0)	2366.4 (1662.6–3368.1)	1554.5 (1182.8–2043.0)	3318.9 (2351.4–4684.4)

Models used $\log_{10}$-transformed concentrations, season as a fixed effect, and residence as a random intercept. Residual-versus-fitted and normal Q–Q plots did not indicate severe violations. A random seasonal slope was not estimated because winter and summer each comprised one month and the residence panel was incomplete across rounds. Season-specific numbers of observations and residences are given in Table S5. Values below the MDL were replaced with one-half of the compound-specific MDL; TPrP was excluded because its detection frequency was 2.21%.

Table S7. Pairwise seasonal concentration ratios and Holm-adjusted *p*-values from the linear mixed-effects models.

Analyte	Global season *p*	Sp/Su	Sp/Au	Sp/Wi	Su/Au	Su/Wi	Au/Wi
TCEP	<0.001	1.55 (0.285)	2.31 (<0.001)	0.72 (0.285)	1.48 (0.285)	0.46 (0.056)	0.31 (<0.001)
TCIPP	0.010	1.09 (1.000)	1.62 (0.007)	1.14 (1.000)	1.48 (0.225)	1.04 (1.000)	0.71 (0.280)
TDCIPP	<0.001	5.70 (<0.001)	10.27 (<0.001)	1.25 (0.581)	1.80 (0.298)	0.22 (0.002)	0.12 (<0.001)
TPhP	0.015	1.28 (0.881)	1.76 (0.066)	0.74 (0.881)	1.37 (0.881)	0.58 (0.478)	0.42 (0.021)
TBP	<0.001	1.42 (0.594)	3.06 (<0.001)	1.33 (0.594)	2.16 (0.027)	0.94 (0.818)	0.43 (0.014)
TBOEP	0.024	1.10 (1.000)	1.83 (0.029)	1.62 (0.154)	1.67 (0.391)	1.48 (0.440)	0.89 (1.000)
TMPP	<0.001	5.19 (<0.001)	1.24 (0.533)	1.83 (0.270)	0.24 (0.003)	0.35 (0.198)	1.47 (0.533)
Σ_8OPFRs	<0.001	1.29 (0.360)	1.96 (<0.001)	0.92 (0.625)	1.52 (0.093)	0.71 (0.360)	0.47 (<0.001)

Each cell gives the model-estimated ratio of the first season to the second season, followed by the Holm-adjusted *p*-value in parentheses. Sp, spring; Su, summer; Au, autumn; Wi, winter. Ratios above 1 indicate higher concentrations in the first-listed season.

Table S8. Key parameters used for retrospective exposure assessment.

Parameter	Infants (1–3 years)	Adults (≥18 years)	Source / note
Body weight (kg)	13.2	63.5	Chinese Exposure Factors Handbooks [32,33]
Dust ingestion rate (mg·day^{-1})	41	30	Chinese Exposure Factors Handbooks [32,33]
Indoor residence fraction	0.875	0.792	USEPA time–activity assumptions
Respiratory rate (breaths·min^{-1})	24	14	Weiss et al. / exposure model
Tidal volume (L·breath^{-1})	0.12	0.48	Weiss et al. / exposure model
Particle concentration, mean (mg·m^{-3})	0.01	0.01	PM2.5 assumption [34]
Particle concentration, high (mg·m^{-3})	0.105	0.105	PM4 assumption [28]
Total deposition fraction	0.61	0.77	MPPD estimates [29]
Exposed skin area (cm^2)	2564	4615	Exposure model [24]
Dust adherence (mg·cm^{-2})	0.04	0.01	Exposure model [24]

Compound-specific gastrointestinal bioaccessibility: TCEP 100%, TCIPP 90.7%, TDCIPP 46.2%, TBP 76.7%, TBOEP 86.2%, TPhP 35.6%, and TMPP 24.0% [25]. Pulmonary bioaccessibility: TCEP 97%, TCIPP 97%, TDCIPP 55%, TPhP 26%, and TMPP 2.3%; 100% was conservatively applied to TBP and TBOEP because compound-specific values were unavailable [26]. Dermal absorption fractions were TCEP 0.28, TCIPP 0.25, TDCIPP 0.13 [27], TBP 0.04 [30], TBOEP 1.0, TPhP 0.5 [31], TMPP 0.5, and TPrP 1.0. No additional oral bioavailability multiplier was applied.

Text S1. Detailed Exposure and Health-Risk Assessment

Human exposure was estimated through dust ingestion, inhalation, and dermal absorption. Calculations reconstruct a screening-level scenario corresponding to measured 2016 concentrations. The 95th-percentile result is a 95th-percentile-concentration scenario, not a reasonable maximum exposure scenario. Uptake-adjusted pathway totals are exposure descriptors and are not directly compared with oral toxicity benchmarks.

S1.1. Dust Ingestion

$EDI_{ing} = (C_{dust} \times DI \times f_{GI} \times t_{home}) / BW$

where EDI_{ing} is the gastrointestinally bioaccessible ingestion dose ($ng \cdot kg^{-1} \cdot day^{-1}$), C_{dust} is the OPFR concentration in dust ($ng \cdot g^{-1}$), DI is the dust ingestion rate ($g \cdot day^{-1}$), f_{GI} is compound-specific gastrointestinal bioaccessibility, t_{home} is the fraction of time spent at home, and BW is body weight (kg) [24,25,32,33]. No additional oral bioavailability factor was applied because independence from f_{GI} could not be established.

S1.2. Inhalation

$EDI_{inh} = (C_{dust} \times P_{inh} \times f_{lung}) / BW$

$P_{inh} = RR \times TV \times 1440 \times t_{home} \times C_p \times DF_{tot} \times 10^{-6}$

where P_{inh} is the deposited dust mass ($g \cdot day^{-1}$), RR is respiratory rate ($breaths \cdot min^{-1}$), TV is tidal volume ($L \cdot breath^{-1}$), C_p is the indoor particle concentration ($mg \cdot m^{-3}$), DF_{tot} is the total pulmonary deposition fraction, and 10^{-6} converts L and mg to m^3 and g. f_{lung} is compound-specific pulmonary bioaccessibility [24,26,28,29,34].

S1.3. Dermal Absorption

$EDI_{derm} = (C_{dust} \times ESS \times DAS \times AF \times t_{home}) / (1000 \times BW)$

where ESS is exposed skin surface area (cm^2), DAS is dust adherence to skin ($mg \cdot cm^{-2}$), AF is the dermal absorption fraction, and the factor 1000 converts mg dust to g dust [24,27,30,31].

S1.4. Total Intake and Non-Carcinogenic Risk

$EDI_{uptake,total} = EDI_{ing} + EDI_{inh} + EDI_{derm}$

$ADI_{oral} = (C_{dust} \times DI \times t_{home}) / BW$

$HQ_{ing} = ADI_{oral} / RfD$

HQ_{ing} values above 1 were considered indicative of potential non-carcinogenic concern. Reference doses were 7000 $ng \cdot kg^{-1} \cdot day^{-1}$ for TCEP, 10,000 for TCIPP, 20,000 for TDCIPP, 10,000 for TBP, 15,000 for TBOEP, 70,000 for TPhP, and 13,000 for TMPP [35,36]. TPrP was excluded because of its 2.21% detection frequency. Inhalation and dermal HQs were not calculated using oral RfDs.

S1.5. Carcinogenic Risk

For TCEP and TBP, $CR = ADI_{oral} \times 10^{-6} \times SFO$, where 10^{-6} converts ng to mg and SFO is the oral cancer slope factor. SFO values were 2×10^{-2} $(mg \cdot kg^{-1} \cdot day^{-1})^{-1}$ for TCEP [12] and 9×10^{-3} $(mg \cdot kg^{-1} \cdot day^{-1})^{-1}$ for TBP from the U.S. EPA PPRTV assessment [37]. For TDCIPP, CR = (oral daily intake / NSRL) × 10^{-5}, using an NSRL of 5.4 $\mu g \cdot day^{-1}$ [38]. These oral screens do not include inhalation, dermal, or mixture risk.

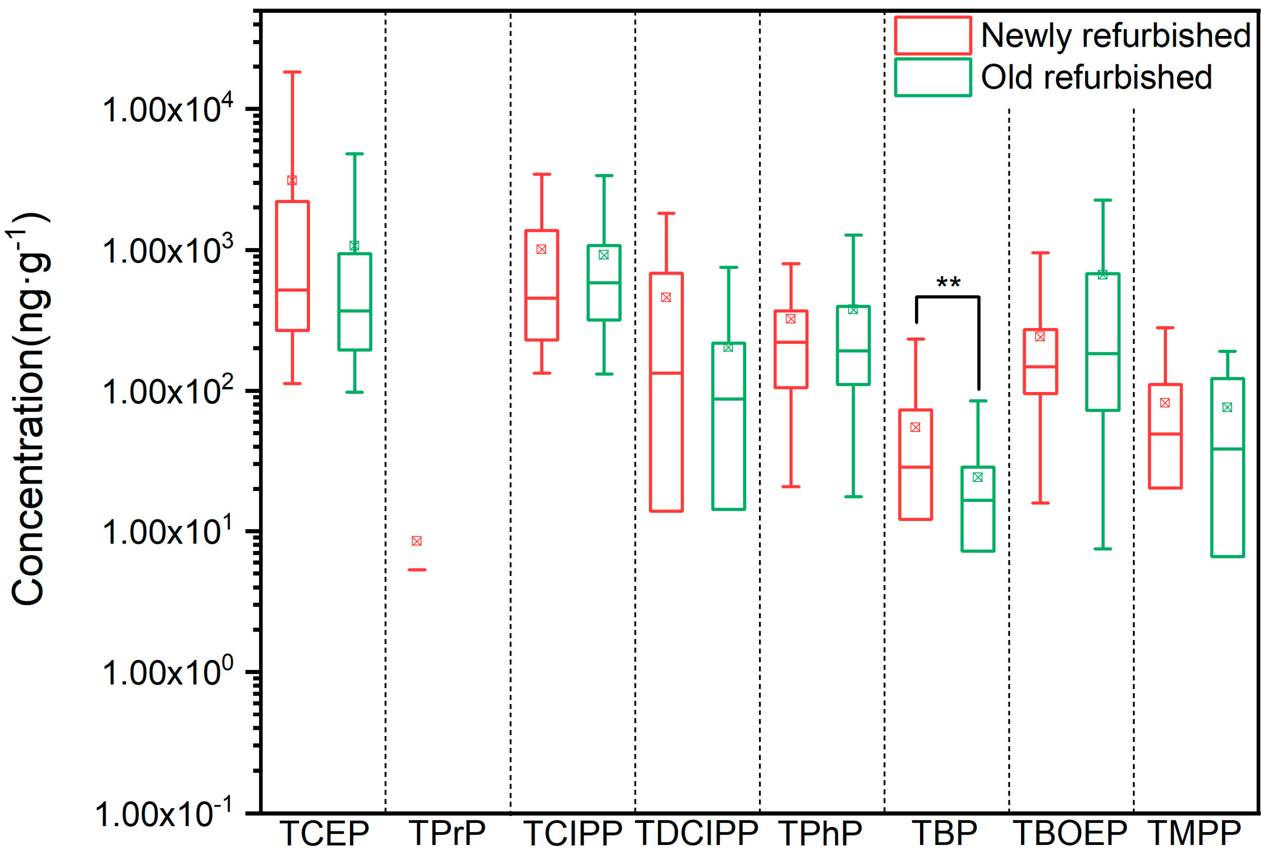

Figure S1. Comparison of OPFR concentrations in dust between newly refurbished homes (refurbished after 2015) and earlier-refurbished homes. ** $p < 0.01$.

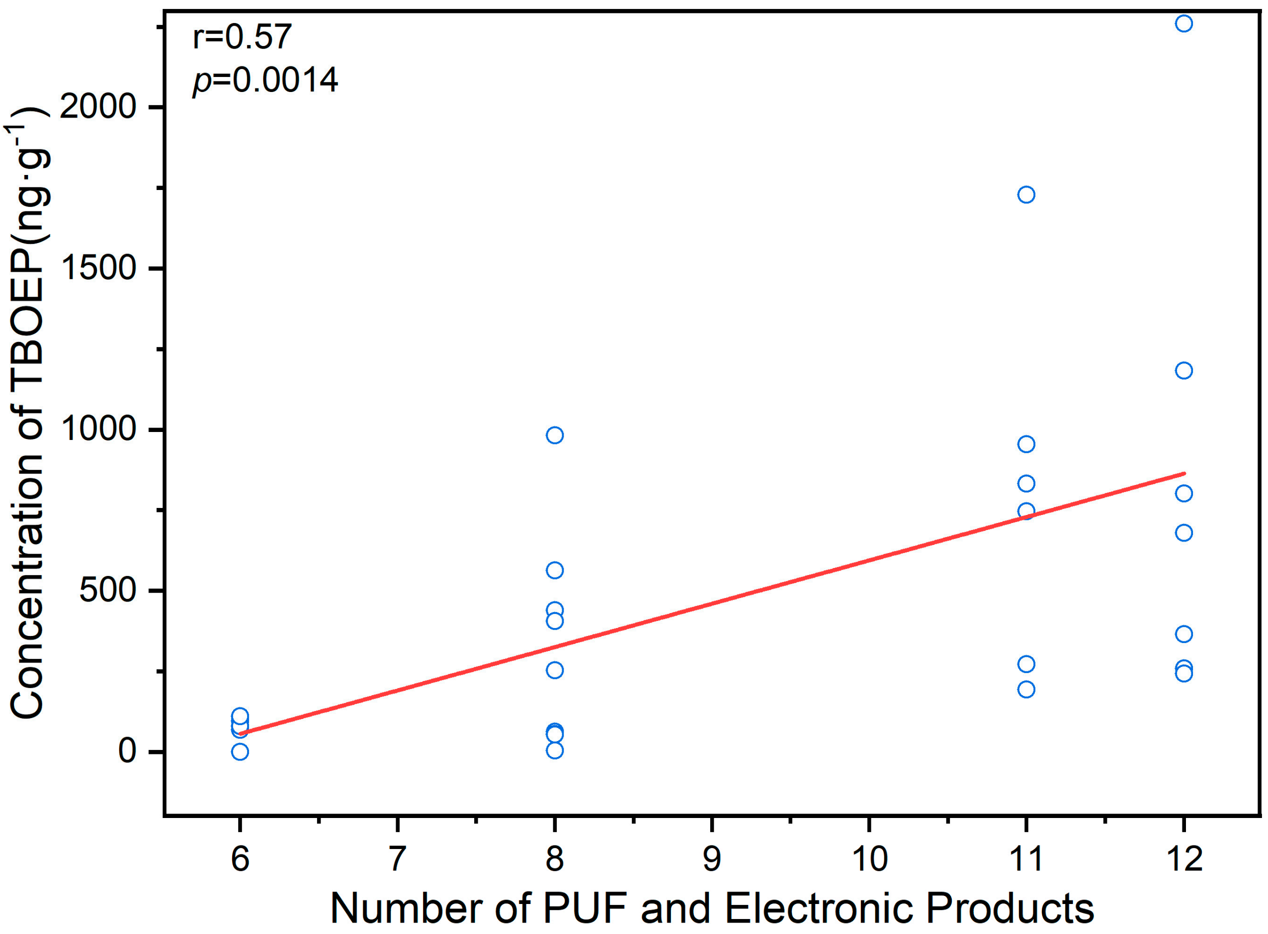

Figure S2. Pearson correlation between TBOEP concentrations in residential dust and the combined number of polyurethane-foam-containing furnishings and electrical/electronic products.

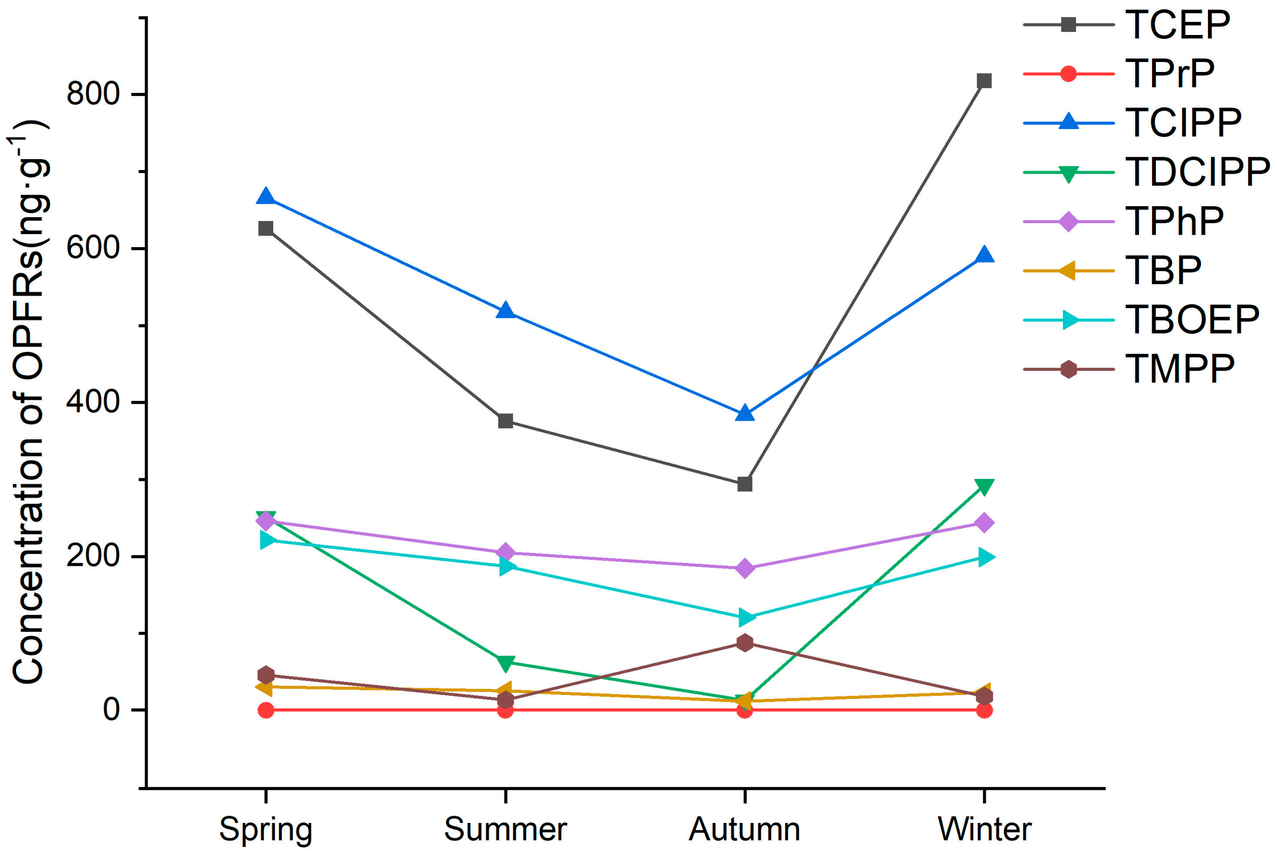

Figure S3. Seasonal trends in median concentrations of OPFR congeners in the year-round residential dust dataset.

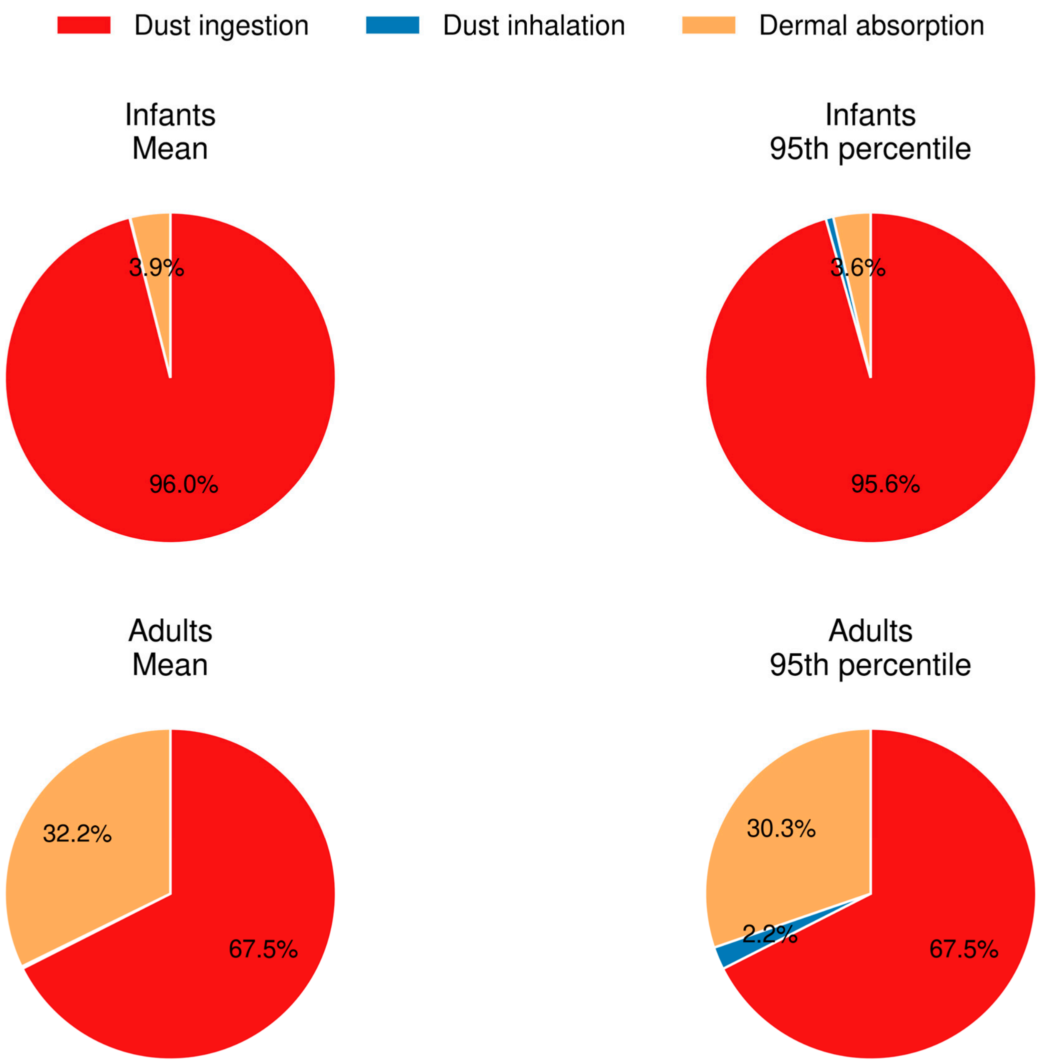

Figure S4. Contributions of ingestion, inhalation, and dermal absorption to uptake-adjusted total EDI in infants and adults under the mean- and 95th-percentile-concentration scenarios.

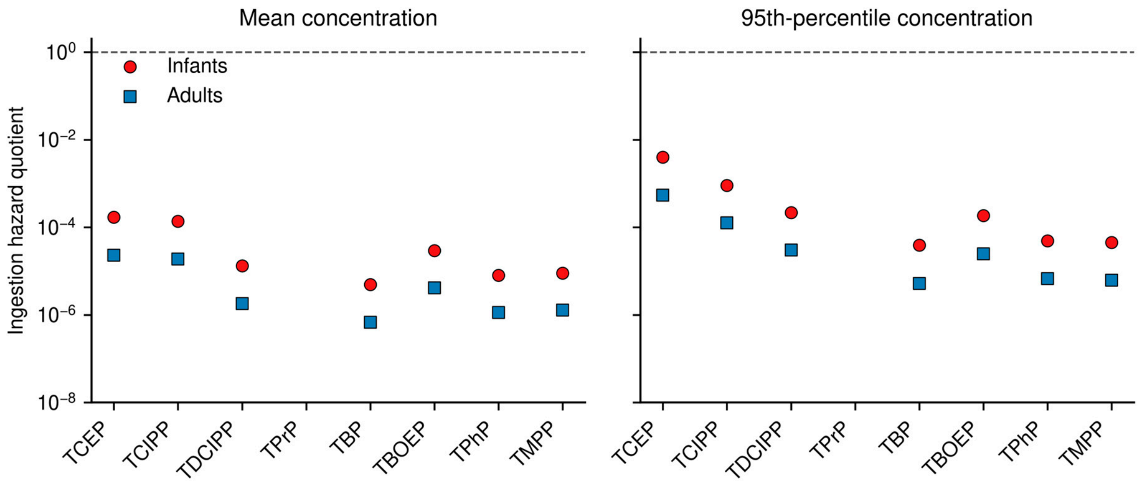

Figure S5. Oral-ingestion hazard quotients calculated from administered dust-ingestion doses for infants and adults under the mean- and 95th-percentile-concentration scenarios. TPrP was excluded because its residential detection frequency was 2.21%.

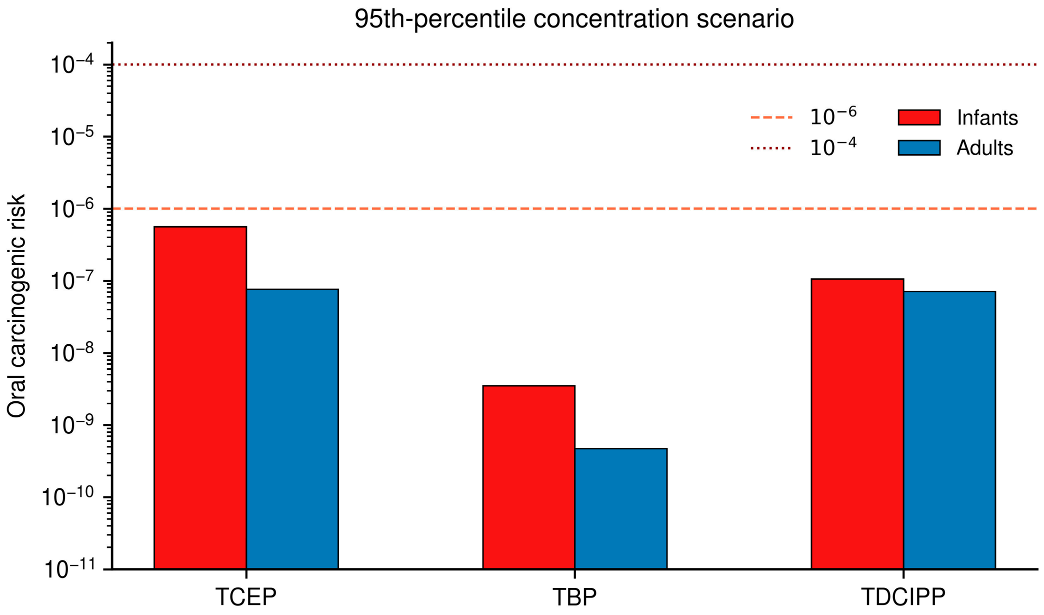

Figure S6. Oral carcinogenic screening values for TCEP, TBP, and TDCIPP in infants and adults under the 95th-percentile-concentration scenario. Dashed lines indicate the 10^{-6} and 10^{-4} screening levels.
